# The Polymer Physics and Theory Section

**DOI:** 10.3390/polym17212832

**Published:** 2025-10-23

**Authors:** Martin Kröger

**Affiliations:** 1 Magnetism and Interface Physics, Department of Materials, ETH Zurich, 8093 Zurich, Switzerland; mk@mat.ethz.ch; 2 Computational Polymer Physics, Department of Materials, ETH Zurich, 8093 Zurich, Switzerland

The section “Polymer Physics and Theory” of *Polymers* has significantly grown in quality since 2023. Apart from the efforts undertaken by the Editorial Board Members, the success of *Polymers* is deeply rooted in the excellent work carried out with the professional attitude of the members of the Editorial and Managing Offices and critical reviewers. As confirmed by the data, published articles are very well received by the scientific community.

The collection of topics presented within this section during the past few years reflects the extraordinary breadth and dynamism of contemporary polymer science, where modeling, theory, synthesis, characterization, and application converge. Across all contributions, a common thread emerges: the effort to bridge molecular-level understanding with macroscopic function, often through sophisticated simulations, experiments, and data-driven approaches. From molecular dynamics and coarse-grained modeling to continuum-scale finite element analysis, polymers are being explored at every relevant scale, enabling a comprehensive multiscale understanding of their structural, mechanical, and dynamic properties.

At the heart of these efforts lies the challenge of complexity. Polymers form entangled, semiflexible, and dynamically crosslinked networks whose behavior under confinement, flow, or external stimuli continues to reveal new physical insights. Theoretical advances in polymer dynamics, rheology, and phase behavior—alongside simulations of entangled melts, gels, and composites—are refining our ability to predict deformation, self-assembly, and transport phenomena. The inclusion of semiflexible systems, biopolymers, and active matter underscores how polymer physics increasingly overlaps with biology and soft matter research, linking the statistical mechanics of chains to the functional behavior of cytoskeletal filaments, membranes, and protein networks.

A parallel frontier has developed around material design and functionality. Smart and adaptive polymers—self-healing, photo- or mechano-responsive, dynamic covalent, or shape-memory—illustrate how chemistry, mechanics, and physics can merge to engineer responsive architectures. Advances in nanocomposites, tribology, and porous polymer structures demonstrate how interfacial control, filler architecture, and topology influence mechanical and multifunctional performance, from thermal management and barrier properties to electrical and tribological behavior. Meanwhile, new emphasis on additive manufacturing, renewable sources, and the circular economy signals a shift toward sustainable polymer innovation and tailored manufacturing pathways.

Computation and theory now underpin nearly every aspect of this landscape. The field moves decisively toward multiscale and multiphysics frameworks, uniting molecular simulation with machine learning, finite element modeling, and experimental data. Artificial intelligence increasingly accelerates materials discovery, process optimization, and structure–property prediction, while advanced simulations of polymerization kinetics, self-assembly, and interfacial phenomena continue to expand our predictive reach. From plasma surface treatments and fiber-reinforced composites to complex liquid crystal systems and polymer-based electronics, these studies embody the evolving role of polymers as enabling materials for technologies that are flexible, adaptive, and sustainable.

To be more specific, we next focus on a few selected contributions. Epoxy resins remain indispensable materials due to their excellent chemical resistance, mechanical strength, and adhesion properties [1,2]. Yet, their high crosslink density after curing results in brittleness and limited toughness. Numerous modification strategies have been explored, including nanofillers (such as graphene, carbon nanotubes, montmorillonite, and silica) and other additives such as thermoplastics and rubbers [3]. Recent studies emphasize the synergistic reinforcement achievable through nanofillers alone, particularly graphene platelets and multiwalled carbon nanotubes [4]. Additive manufacturing (AM) further broadens the scope of polymer processing [5], though challenges persist regarding residual stress and warpage deformation [6]. Thermal gradients in fused filament fabrication significantly affect polymer rheology and mechanical response, as demonstrated by systematic experimental analyses of reinforcement direction, density, and processing parameters [7,8].

Other developments highlight the growing impact of advanced and functional polymers. Liquid crystal polymers (LCPs) combine anisotropic order with polymeric flexibility, enabling applications in optoelectronics, sensors, membranes, and fuel cells [9,10,11]. Computational modeling (CM) has become a powerful tool for designing biopolymers, integrating computer-aided design, finite element analysis, and molecular dynamics simulations to bridge in silico and in vitro approaches [12,13,14]. Emerging polymer classes such as covalent adaptable networks and vitrimers exhibit reversible bond exchange and offer recyclability and self-healing capabilities [15,16,17]. Biodegradable materials like poly(lactic acid) (PLA) are being optimized through plasticization with poly(ethylene glycol) to enhance flexibility [18,19,20]. Machine learning now accelerates polymer design, extracting structure–property relationships from large datasets to predict key parameters such as glass transition temperature [21,22]. Finally, polymer-based functional films—such as cellulose triacetate modified with carbon nanotubes—demonstrate high sensitivity and reproducibility in detecting environmental pollutants at trace levels [23,24,25].

In summation, the section and its numerous Special Issues capture a field in transformation—one where physics, chemistry, computation, and engineering cooperate to design polymeric materials that respond, heal, assemble, and perform across scales and environments. The interplay between modeling and experiment, function and form, and sustainability and innovation defines not only the current state of polymer research but also its expanding horizons.
